# Blood Glucose Meter Buying Behavior of Diabetic Patients: Factors Influencing Purchase

**DOI:** 10.3389/fpubh.2022.880088

**Published:** 2022-05-16

**Authors:** Bai-ling Pan, Yi-tong Pan, Zu-hua Gao, Tao-Hsin Tung

**Affiliations:** ^1^Department of Endocrinology, Taizhou Hospital of Zhejiang Province, Wenzhou Medical University, Linhai, China; ^2^Evidence-Based Medicine Center, Taizhou Hospital of Zhejiang Province, Wenzhou Medical University, Linhai, China

**Keywords:** type 2 diabetes mellitus, glucose meter, buying behavior, insulin therapy, self-monitoring of blood glucose, influencing factors

## Abstract

**Objective:**

To understand the blood glucose meter buying behavior of type 2 diabetic patients with poor glycemic control (two or more HbA1c ≥ 8% during visits in one year) and identify factors influencing it.

**Methods:**

A survey was conducted among 585 diabetic patients with poor glycemic control who were treated in the outpatient or inpatient clinics of the Department of Endocrinology, Taizhou Hospital, Zhejiang Province from June 2020 to May 2021. The questionnaire collected general information and clinical data, and assessed blood glucose meter buying behavior. Chi-square test was used to compare the essential characteristics and clinical data between buyers and non-buyers of blood glucose meters. Additionally, stepwise logistic regression was used to analyze the factors influencing purchase.

**Results:**

Of the 585 questionnaires distributed, 527 (90.09%) valid questionnaires were collected. Of the 527 respondents, 285 (54.08%) had purchased blood glucose meters. Not receiving insulin therapy (OR: 1.77, 95% CI: 1.13–2.77) and unawareness of self-monitoring of blood glucose (OR: 19.46, 95% CI: 12.51–30.26) were risk factors for non-purchase.

**Conclusion:**

There is a need to actively increase the purchase of glucose meters among diabetic patients, by educating them about the importance of self-monitoring of blood glucose.

## Introduction

Diabetes is a chronic metabolic disease caused by genetic and environmental factors. The 2017 International Diabetes Federation (IDF) report showed that China had the highest number of diabetes patients globally (1). According to the American Diabetes Association (ADA) (2), 12.8% of Chinese adults (estimated 129.8 million−70.4 million men and 59.4 million women) were diagnosed with diabetes. The diabetes awareness rate was 43.3%, the treatment rate was 49.0%, and the control rate was 49.4%, making diabetes one of China's crucial public health issues. After contracting the disease, the body would be high in glucose, inducing a series of serious complications. The Diabetes Control and Complications Trial (DCCT) in the US and the United Kingdom Prospective Diabetes Study (UKPDS) have shown that controlling blood glucose was an effective measure for reducing the incidence of diabetic complications (3, 4). The key to glycemic control was the patient's daily behavior and the capacity for self-management. With the development of medical treatment, the systematic management of the diabetes system has become more important than treatment in the traditional sense (5).

As an essential part of diabetes management, blood glucose monitoring is the standard practice through which physicians adjust clinical treatment methods and try to minimize late complications. There are several clinical methods used for blood glucose monitoring for the daily management of diabetes, among which capillary blood glucose monitoring using a glucose meter is an essential and fundamental tool (6). The world's first pocket-sized blood glucose meter was introduced in the late 1960s, measuring blood glucose through a drop of capillary blood. This meter has the advantages of being small, easy to operate, home use friendly (as very little blood is drawn and there is no requirement for intravenous blood collection), fast, and economical (7). Scholars (8) have compared the blood glucose values obtained by the two blood glucose meters and automatic biochemical analyzers tests and found a good correlation between the measured values. However, blood glucose meter usage varies due to differences in the level of economic development and availability of diabetes treatment in different parts of China (6). This study aimed to understand the overall trend in the purchase of blood glucose meters by diabetic patients and identify the factors influencing the purchase in order to provide a basis for the possession rate of blood glucose meters by diabetic patients.

## Subjects and Methods

### Study Subjects

Participants were 585 diabetes patients with poor glycemic control (two or more HbA1c ≥ 8% in visits during 1 year) discharged after outpatient or inpatient treatment from the Department of Endocrinology, Taizhou Hospital of Zhejiang Province from June 2020 to May 2021. All study subjects met the 1999 WHO diagnostic criteria for diabetes mellitus (9), had no communication obstacles. This study was exempted from informed consent and was approved by the Ethics Committee of Taizhou Hospital of Zhejiang Province (approval number: K20211215) in China. All procedures were performed in accordance with the guidelines of our institutional ethics committee and adhered to the tenets of the Declaration of Helsinki. All participants' information was maintained anonymously.

### Research Methodology

#### Survey Instruments

A questionnaire on the factors influencing the purchase of blood glucose meters by diabetic patients was independently developed after discussions and modifications by nursing experts. The questionnaire collected demographic and general information (gender, age, residence, education, occupation, knowledge of glycemic control goals, understanding of self-monitoring of blood glucose [SMBG], and purchase of blood glucose meters). Further, including the study subjects' clinical characteristics—duration of diabetes, use of insulin therapy, previous episodes of hypoglycemia, chronic complications of diabetes, and the presence of hypertension or hyperlipidemia, or both.

#### Survey Methodology

This was a prevalence survey, and two endocrine nurses collected data after unified training. The study ascertained the subjects' right to informed consent and the principle of voluntariness, and the questionnaires were collected face-to-face. The study purpose was explained to the study subjects before the survey, and the method of questionnaire completion was also explained. The study subjects completed the questionnaires independently, after which the investigator collected them.

### Statistical Handling

The SPSS Windows software version 25.0 was used for statistical analysis. Count data were expressed in several cases, and ratio (%) and χ^2^ test were used for inter-group comparison. Stepwise logistic regression was used to analyze the factors influencing the purchase of blood glucose meters by diabetic patients. A test level α = 0.05 was adopted.

## Results

### Purchase of Blood Glucose Meters by Diabetic Patients

We distributed 585 questionnaires. Of these, 527 valid questionnaires were recovered, with a valid recovery rate of 90.09%. Analysis of the 527 valid questionnaires showed that 285 subjects (54.08%) had purchased blood glucose meters and 242 subjects (45.92%) had not purchased them.

### Univariate Analysis of Glucose Meter Purchase Among Populations With Different Characteristics

Statistical differences were observed between patients who purchased glucose meters and those who did not—in terms of education, occupation, the use of insulin therapy, previous episodes of hypoglycemia, chronic complications of diabetes, knowledge of glucose control goals, and the knowledge of SMBG (*P* < 0.05). Among patients who purchased blood glucose meters, the percentages for the following variables were lower than those who did not purchase blood glucose meters (Table 1): educational level of primary school or below, *P* = 0.001; being a farmer, *P* < 0.01; no use of insulin therapy, *P* = 0.001; no previous episodes of hypoglycemia *P* = 0.003; no chronic complications of diabetes, *P* = 0.013; no understanding of blood glucose control goals, *P* = 0.013; and no understanding of SMBG, *P* < 0.01.

**Table 1:** Univariate analysis of purchase of blood glucose meters among populations with different characteristics [*n* (%)].

**Variable**	**Having purchased a blood glucose meter (*n* = 285)**	**No blood glucose meter purchased** **(*n* = 242)**	**χ^2^**	***P***
Gender				
Male	155 (54.4)	128 (52.9)	0.12	0.73
Female	130 (45.6)	114 (47.1)		
Age group (years)				
<60	159 (55.8)	152 (62.8)	2.67	0.10
≥ 60	126 (44.2)	90 (37.2)		
Place of residence				
Rural	164 (57.5)	156 (64.5)	2.63	0.11
Non-rural	121 (42.5)	86 (35.5)		
Educational level				
Primary school and below	129 (45.3)	146 (60.3)	11.91	0.001
Junior middle school and above	156 (54.7)	96 (39.7)		
Occupation				
Farmer	132 (46.3)	150 (62.0)	12.91	<0.01
Non-farmer	153 (53.7)	92 (38.0)		
The course of diabetes (years)				
≤ 10	162 (56.8)	151 (62.4)	1.67	0.20
> 10	123 (43.2)	91 (37.6)		
Insulin therapy				
Yes	183 (64.2)	119 (49.2)	12.10	0.001
No	102 (35.8)	123 (50.8)		
Hypoglycemia				
Yes	143 (50.2)	90 (37.2)	8.95	0.003
No	142 (49.8)	152 (62.8)		
Hypertension				
Yes	125 (43.9)	96 (39.7)	0.94	0.33
No	160 (56.1)	146 (60.3)		
Hyperlipidemia				
Yes	72 (25.3)	73 (30.2)	1.58	0.21
No	213 (74.7)	169 (69.8)		
Chronic complications of diabetes				
Yes	123 (43.2)	79 (32.6)	6.12	0.013
No	162 (56.8)	163 (67.4)		
Knowledge of blood glucose control goals				
Yes	144 (50.5)	96 (39.7)	6.22	0.013
No	141 (49.5)	146 (60.3)		
Knowledge of SMBG				
Yes	239 (83.9)	51 (21.1)	208.47	<0.01
No	46 (16.1)	191 (78.9)		

### Factors Influencing the Purchase of Blood Glucose Meters by Diabetic Patients

The purchase of a blood glucose meter was used as a dependent variable to further analyze the effect of education level, occupation, the use of insulin therapy, previous episodes of hypoglycemia, chronic complications of diabetes, knowledge of blood glucose control goals, and knowledge of SMBG on not purchasing a blood glucose meter. This analysis was conducted using a stepwise logistic regression model. The results showed that no use of insulin therapy (OR: 1.77, 95% CI: 1.13–2.77) and unawareness of SMBG (OR: 19.46, 95% CI: 12.51–30.26) were risk factors for not purchasing a blood glucose meter in diabetic patients as shown in Table 2.

**Table 2:** Stepwise logistic regression analysis on factors affecting the purchase of blood glucose meters by diabetic patients.

**Variable**	**Category**	**β**	**SE**	***P***	**OR (95% CI)**
Insulin therapy	Yes vs. No	0.57	0.23	0.013	1.77 (1.13–2.77)
Knowledge of SMBG	Yes vs. No	2.96	0.23	<0.01	19.46 (12.51–30.26)

*Control variables: education level, occupation, previous episodes of hypoglycemia, chronic complications of diabetes, and knowledge of glycemic control goals*.

## Discussion

When a patient is diagnosed with diabetes, it is recommended that they use a blood glucose meter on a routine basis. However, in this study, the glucose meter buying behavior of diabetic patients was considerably pessimistic. The results showed that the number of glucose meter purchases was lower in patients who are not on insulin therapy than those receiving insulin therapy (*p* = 0.013). Mariye et al. (10) found that having a glucose meter at home was positively associated with adherence to insulin therapy. This could be because diabetic patients generally believed that they only needed to monitor their blood glucose on insulin therapy (11). Patients on insulin therapy may have also received education on blood glucose monitoring when they were educated on insulin use. For patients on insulin therapy, most current guidelines recommend glucose monitoring at least three times a day (12). Therefore, clinical practice must focus on providing education on glucose monitoring to patients not receiving insulin therapy, mainly to make them follow guidelines related to glucose monitoring and help them become aware of the importance of glucose monitoring to increase the possession rate of glucose meters.

This study also established that patients aware of SMBG were more willing to purchase blood glucose meters, and the difference was statistically significant (*p* < 0.01). Conversely, one study found that many patients were unclear about SMBG recommendations, faced practical testing barriers, and were unsure how to integrate SMBG into their lives (13). All guidelines published by the IDF, ADA, and the National Institute for Health and Clinical Excellence (NICE) emphasized that SMBG was recommended for all diabetic patients (6). Standard self-monitoring was an essential means of achieving blood glucose goals for diabetic patients, which provided effective control of blood glucose and helped to improve glycosylated hemoglobin levels in people with type 2 diabetes who were not on insulin therapy (14, 15). A blood glucose meter is an essential tool for SMBG, which can reflect real-time blood glucose status, help patients better understand the state of their disease, and motivate them to actively manage their diabetes. It can help improve treatment adherence and reduce diabetes mortality and disability. Therefore, the advantages of SMBG should be highlighted during diabetes health education, which can be done in various forms such as lectures, distribution of promotional materials, support groups or clubs, glucose patient clubs, multimedia, WeChat support, and follow-up visits (16–18). The “Chinese clinical application guidelines for glucose monitoring (2015 edition) (6)” may be referenced for specific measures to formulate the glucose monitoring plan based on the patient's treatment method (lifestyle intervention, oral hypoglycemic drugs, and insulin therapy, etc.). The corresponding blood glucose monitoring protocol book (with the interpretation of blood glucose values) should be designed and distributed among the patients for blood glucose recording to improve patients' enthusiasm for blood glucose monitoring and handle blood glucose values correctly.

This was a cross-sectional study, and no conclusions on the exact causality can be drawn yet. However, it is also possible that patients who had a glucose meter were more aware of self-monitoring. Moreover, only diabetic patients with poor glycemic control were investigated, with limited representativeness. The factors for the unsatisfactory glucose meter possession among diabetic patients included: whether they were on insulin therapy and were aware of SMBG. Therefore, it is of utmost urgency for diabetes educators to: teach diabetic patients, especially patients on oral hypoglycemic therapy, about SMBG; emphasize the importance of owning a blood glucose meter; and guide standardized blood glucose monitoring.

## Data Availability Statement

The original contributions presented in the study are included in the article/supplementary material, further inquiries can be directed to the corresponding authors.

## Author Contributions

T-HT and Z-hG conceived the study. B-lP, Y-tP, and T-HT designed the questionnaire. B-lP and Y-tP collected the data. B-lP, T-HT, and Z-hG analyzed and interpreted the data and wrote the first draft of the paper. Y-tP searched, sorted, and interpreted the relevant literature. All authors edited and approved the final manuscript.

## Conflict of Interest

The authors declare that the research was conducted in the absence of any commercial or financial relationships that could be construed as a potential conflict of interest.

## Publisher's Note

All claims expressed in this article are solely those of the authors and do not necessarily represent those of their affiliated organizations, or those of the publisher, the editors and the reviewers. Any product that may be evaluated in this article, or claim that may be made by its manufacturer, is not guaranteed or endorsed by the publisher.
